# Neuropsychiatric Symptom Burden across Neurodegenerative Disorders and its Association with Function

**DOI:** 10.1177/07067437221147443

**Published:** 2023-01-13

**Authors:** Daniel Kapustin, Shadi Zarei, Wei Wang, Malcolm A. Binns, Paula M. McLaughlin, Agessandro Abrahao, Sandra E. Black, Michael Borrie, David Breen, Leanna Casaubon, Dar Dowlatshahi, Elizabeth Finger, Corinne E Fischer, Andrew Frank, Morris Freedman, David Grimes, Ayman Hassan, Mandar Jog, Donna Kwan, Anthony Lang, Brian Levine, Jennifer Mandzia, Connie Marras, Mario Masellis, Joseph B. Orange, Stephen Pasternak, Alicia Peltsch, Bruce G. Pollock, Tarek K. Rajji, Angela Roberts, Demetrios Sahlas, Gustavo Saposnik, Dallas Seitz, Christen Shoesmith, Alisia Southwell, Thomas D.L. Steeves, Kelly Sunderland, Richard H Swartz, Brian Tan, David F. Tang-Wai, Maria Carmela Tartaglia, Angela Troyer, John Turnbull, Lorne Zinman, the ONDRI investigators, Sanjeev Kumar

**Affiliations:** 1 7938 Department of Psychiatry, University of Toronto, Toronto, ON, Canada; 2 7978 Adult Neurodevelopmental and Geriatric Psychiatry Division, Centre for Addiction and Mental Health, Toronto, ON, Canada; 3 Dalla Lana School of Public Health, 7938 University of Toronto, Toronto, ON, Canada; 4 Rotman Research Institute at Baycrest Health Sciences, Toronto, ON, Canada; 5 432234 Nova Scotia Health, Halifax, Nova Scotia, Canada; 6 Division of Neurology, Department of Medicine, University of Toronto, Ontario, Canada; 7 Division of Neurology, Department of Medicine, 71545 Sunnybrook Health Sciences Centre, Toronto, ON, Canada; 8 Department of Medicine (Neurology), 7938 University of Toronto, Toronto, ON, Canada; 9 LC Campbell Cognitive Neurology Unit, Hurvitz Brain Sciences Program, Sunnybrook Research Institute, 7938 University of Toronto, Toronto, ON, Canada; 10 University of Western Ontario, London, ON, Canada; 11 Centre for Clinical Brain Sciences, University of Edinburgh, Edinburgh, UK; 12 Department of Medicine (Neurology), University of Ottawa Brain and Mind Institute and Ottawa Hospital Research Institute, Ottawa, ON, Canada; 13 Keenan Research Centre for Biomedical Science, St. Michaels Hospital, Toronto, Ontario, Canada; 14 6363 University of Ottawa, Ottawa, ON, Canada; 15 152971 Bruyere Research Institute, Ottawa, ON, Canada; 16 Thunder Bay Regional Research Institute, 26627 Northern Ontario School of Medicine, ON, Canada; 17 4257 Queen's University, Kingston, ON, Canada; 18 Edmond J. Safra Program in Parkinson's Disesase and the Morton and Gloria Shulman Movement Disorders Centre, University Health Network, Toronto, ON, Canada; 19 Canadian Centre for Activity and Aging, Western University, ON, Canada; 20 Robarts Research Institute and the Department of Medical Biophysics, The University of Western Ontario, London, ON, Canada; 21 Dementia Research Alliance, University of Toronto, Toronto, Ontario, Canada; 22 School of Communication, Northwestern University, Evanston, Illinois, USA; 23 School of Communication Sciences and Disorders, Western University, London, Ontario, Canada; 24 3710 McMaster University, Hamilton, ON, Canada; 25 Department of Psychiatry and Hotchkiss Brain Institute, Cumming School of Medicine, University of Calgary, Calgary, AB, Canada; 26 Division of Neurology, Department of Clinical Neurological Sciences, London Health Sciences Centre, London, ON, Canada; 27 71545 Sunnybrook Health Sciences Centre, Toronto, ON, Canada; 28 Krembil Brain Institute, Toronto Western Hospital, Toronto, ON, Canada; 29 Toronto Western Hospital, University Health Network Memory Clinic, Toronto, ON, Canada; 30 Tanz Centre for Research in Neurodegenerative Diseases, 7938 University of Toronto, Toronto, ON, Canada; 31 Neuropsychology and Cognitive Health Program, Baycrest Health Sciences, Toronto, ON, Canada

**Keywords:** aging, aging and memory, Alzheimer's disease, cohort study, geriatric psychiatry

## Abstract

**Objective:**

Neuropsychiatric symptoms (NPS) are prevalent in neurodegenerative disorders, however, their frequency and impact on function across different disorders is not well understood. We compared the frequency and severity of NPS across Alzheimer's disease (AD) (either with mild cognitive impairment or dementia), Cerebrovascular disease (CVD), Parkinson's disease (PD), frontotemporal dementia (FTD), and amyotrophic lateral sclerosis (ALS), and explored the association between NPS burden and function.

**Methods:**

We obtained data from Ontario Neurodegenerative Disease Research Initiative (ONDRI) that included following cohorts: AD (*N* = 111), CVD (*N* = 148), PD (*N* = 136), FTD (*N* = 50) and ALS (*N* = 36). We compared the frequency and severity of individual NPS (assessed by the neuropsychiatric inventory questionnaire) across cohorts using generalized estimating equations and analysis of variance. Second, we assessed the relationship of NPS burden with instrumental (iADLs) and basic (ADLs) activities of living across cohorts using multivariate linear regression while adjusting for relevant demographic and clinical covariates.

**Results:**

Frequency of NPS varied across cohorts (χ^2^_(4)_ = 34.4, *p* < .001), with post-hoc tests showing that FTD had the greatest frequency as compared to all other cohorts. The FTD cohort also had the greatest severity of NPS (*H*_(4)_ = 34.5, *p* < .001). Further, there were differences among cohorts in terms of the association between NPS burden and ADLs (*F*_(4,461)_ = 3.1, *p* = 0.02). Post-hoc comparisons suggested that this finding was driven by the FTD group, however, the differences did not remain significant following Bonferroni correction. There were no differences among cohorts in terms of the association between NPS burden and IADLs.

**Conclusions:**

NPS frequency and severity are markedly greater in FTD as compared to other neurodegenerative diseases. Further, NPS burden appears to be associated differently with function across neurodegenerative disorders, highlighting the need for individualized clinical interventions.

## Résumé

**Objectif:**

Les symptômes neuropsychiatriques (SNP) sont prévalents dans les troubles neurodégénératifs, cependant, leur fréquence et leur effet sur la fonction par différents troubles ne sont pas bien compris. Nous avons comparé la fréquence et la gravité des SNP dans la maladie d’Alzheimer (MA) (soit avec une faible déficience cognitive ou la démence), la maladie cérébrovasculaire (MCV), la maladie de Parkinson (MP), la démence fronto-temporale (DFT), et la sclérose latérale amyotrophique (SLA), et nous avons exploré l’association entre le fardeau des SNP et la fonction.

**Méthodes:**

Nous avons obtenu des données de l’Initiative ontarienne de recherche sur les maladies neurodégénératives (ONDRI) qui comportait les cohortes suivantes : MA (N = 111), (MCV (N = 148), MP (N = 136), DFT (N = 50) et SLA (N = 36). Nous avons comparé la fréquence et la gravité des SNP individuels (évalués par le questionnaire de l’inventaire neuropsychiatrique) dans les cohortes à l’aide d’équations d’estimation généralisée et d’analyse de variance. Ensuite, nous avons évalué la relation du fardeau des SNP avec les activités instrumentales (iADLs) et de base (ADLs) de la vie quotidienne dans les cohortes à l’aide de la régression linéaire multivariée tout en ajustant pour les covariables démographiques et cliniques appropriées.

**Résultats:**

La fréquence des SNP variait selon les cohortes (χ^2^_(4)_ = 34,4, p < ,001), les tests post-hoc indiquant que la DFT avait la plus grande fréquence comparativement à toutes les autres cohortes. La cohorte DFT avait aussi la gravité la plus grande de SNP *(H*_*(* *4* *)*_*= 34,5, p < ,001)*. En outre, il y avait des différences parmi les cohortes en ce qui concerne l’association entre la charge de SNP et les ADL (F_(4,461)_ = 3,1, p = 0,02). Les comparaisons post-hoc suggéraient que ce résultat était amené par le groupe DFT, toutefois, les différences ne sont pas demeurées significatives suite à la correction de Bonferroni. Il n’y avait pas de différences parmi les cohortes en ce qui concerne l’association entre la charge des SNP et les IADL.

**Conclusions:**

La fréquence et la gravité des SNP sont plus grandes de façon marquée dans les DFT si l’on compare à d’autres maladies neurodégénératives. De plus, la charge des SNP semble être associée différemment à la fonction dans les troubles neurodégénératifs, ce qui fait ressortir le besoin d’interventions cliniques individualisées.

## Introduction

Neuropsychiatric symptoms (NPS) constitute a major challenge for patients with Alzheimer's disease (AD), cerebrovascular disease (CVD), Parkinson's disease (PD), frontotemporal dementia (FTD), and amyotrophic lateral sclerosis (ALS).^1,5^ NPS commonly include apathy, depression, anxiety, irritability, agitation, aggression, delusions, hallucinations, as well as sleep and appetite disturbances.^1,5^ NPS occur almost invariably at some point during the course of neurodegenerative illness. Among patients with dementia, the 5-year period prevalence of at least one symptom has been estimated to be up to 97%, with the most common being depression, anxiety, and apathy.^6^ Moreover, the presence of these symptoms has been associated with accelerated disease progression, reduced quality of life, and greater disability.^1,7^

Interestingly, the prevalence of particular neuropsychiatric symptoms differs between specific neurodegenerative disease cohorts.^2,8,9^ Varied symptom presentations across cohorts also pose a significant challenge in the evaluation and management of NPS, as treatment needs to be individualized.^10^ Importantly, NPS has been associated with cognitive impairment, with a higher symptom burden predictive of poorer cognitive function.^11,12^ Specifically, depression and sleep disturbances in AD, while delusions and abnormal eating behaviours in FTD, were negatively associated with cognition.^12^ Moreover, NPS in patients with AD and CVD have been associated with impairments in activities of daily living (ADLs).^8,13,14^ However, most studies lack participant stratification into specific neurodegenerative disease cohorts.^13^ Thus, data assessing NPS and their association with function across diverse, well-defined neurodegenerative disease cohorts is needed to better understand this relationship.^15^

The primary objective of this study was to compare the burden of NPS among AD, CVD, PD, FTD, and ALS cohorts. We hypothesized that these groups will have distinct NPS frequency and severity. We further aimed to explore the association between NPS and function in each cohort while adjusting for demographic and other clinical factors.

## Methods

Data used in this study are from Phase 1 of the Ontario Neurodegenerative Disease Research Initiative (ONDRI) – a longitudinal observational cohort study addressing the phenotypic sequelae of degenerative cognitive impairment. The sample included participants with AD (mild cognitive impairment (MCI) or dementia due to AD), CVD, PD, FTD, or ALS. Detailed inclusion/exclusion criteria of ONDRI participants were reported previously.^16,17^ For MCI, participants with MCI only due to AD were included and they met the National Institute on Aging-Alzheimer's Association (NIA-AA) core clinical criteria for amnestic single or multiple domain mild cognitive impairment.^18^ Other causes of cognitive impairment were ruled out by standardized work up, including brain imaging.^16^ All participants underwent comprehensive evaluations to assess cognition, function, and neuropsychiatric symptoms. Of the enrolled participants, only those providing neuropsychiatric symptom score, and one of either basic or instrumental activities of daily living score described below were included in analyses.

### Neuropsychiatric Symptoms and Cognition

The neuropsychiatric inventory questionnaire (NPI-Q) was used to assess neuropsychiatric symptoms.^19^ Study partners of participants reported on the presence and severity of 12 neuropsychiatric symptoms including delusions, hallucinations, agitation/aggression, depression/dysphoria, anxiety, elation/euphoria, apathy/indifference, disinhibition, irritability/lability, motor disturbances, nighttime behaviours, and appetite/eating. Total scores for symptom severity (maximum of 36) and partner distress (maximum of 60) were computed by adding the individual symptom scores.^20^ Montreal cognitive assessment (MoCA) was used to assess cognition.^21^

### Function

Lawton–Brody instrumental activities of daily living (iADL) scale was used to measure participants’ ability to function independently on instrumental activities of daily living. Study partners rated the participants’ ability across eight iADL tasks, ranging from totally independent to totally dependent, including telephone use, shopping, food preparation, housekeeping, laundering, use of transportation, managing medications, and financial management, for a maximum possible total score of 23.^22^ In the event that a given participant did not engage in an activity pre-morbidly, the activity's score was deducted from that participant's total possible score (maximum of 23). This produced a modified total score for each participant, by which each participant's score was divided to compute an iADL percent score.

The physical self-maintenance scale was used to measure participants’ ability to function independently on basic activities of daily living (ADLs). Study partners rated the participants’ ability to function independently across six basic ADLs: feeding, dressing, grooming, ambulation, bathing, and toileting, for a maximum possible total score of 24.^23^ ADL percent scores were computed for each participant by dividing their total score by 24.

### Motor Function

The ALS functional rating scale-revised (ALSFRS-R) was used in the ALS cohort,^24^ and the Unified Parkinson's Disease Rating Scale (MDS-UPDRS, part III) was used in the PD cohort to assess motor symptoms.^25^

### Statistical Analyses

Statistical analyses were performed using SPSS (IBM Corp, SPSS v25). Chi-square or Fisher's exact test (expected cell count less than five) were used to compare categorical measures among cohorts. Standardized residuals (*z* scores) were evaluated for significant outcomes. For all continuous outcomes, means and standard deviations were computed, normality was assessed using the Shapiro–Wilk test and by visual inspection of data histograms and quantile–quantile plots. Generalized estimating equations (GEE) were used to compare NPS frequency among cohorts, by evaluating binary outcomes (presence of individual NPI-Q symptoms) for individuals within each cohort while accounting for correlation of multiple symptoms measure from the same individual. The GEE covariance structure was specified as “unstructured” as the structure of the within-subject covariance was unknown. One-way analysis of variance (ANOVA) was used to compare NPS severity among cohorts. As applicable, Kruskal–Wallis test was used for non-normally distributed continuous data. Post hoc analyses were completed using Bonferroni testing. To investigate the relationships among NPI-Q total score, MoCA and ADL/iADL scores, correlations were computed using Pearson's or Spearman's correlation coefficient as appropriate, first across combined patient cohorts and further within each cohort. To explore whether the dependence of ADL and iADL percent scores on NPI-Q differed between cohorts, we carried out multivariable linear regression with ADL or iADL score as the dependent variable, NPI-Q and cohort membership as the independent variables with an interaction term, and with age, education, and MoCA as additional independent variables. Subsequently, to explore the association between NPS and function within each cohort, independent of demographic variables and cognition, linear regression was performed with total ADL or iADL percent scores as the dependent variables and NPI-Q total score, age, education, and MoCA scores as the independent variables. AD cohort participants were divided into two groups (MCI or dementia) and analyzed according to diagnosis at the time of study initiation. ALSFRS-R and MDS-UPDRS were included as covariates to control for motor disease burden in ALS and PD cohorts. Bootstrapping using 1,000 samples was performed with all linear regressions to improve interpretability independent of distribution assumptions. Only participants with complete data across measures of interest were included in regressions. Statistical significance was set at *α* = 0.05.

## Results

ONDRI participant cohorts included AD (*n* = 126), CVD (*n* = 161), PD (*n* = 140), FTD (*n* = 53), and ALS (*n* = 40). In the AD cohort, 85 (67.4%) participants were diagnosed with MCI while 41 (32.5%) were diagnosed with dementia. In the FTD cohort, 22 (41.5%) were diagnosed with behavioural variant FTD (bvFTD), 15 (28.3%) with Progressive Supranuclear Palsy (PSP), eight (15.1%) with Primary Progressive Aphasia (PPA), five (9.4%) with Semantic Dementia, and three (5.6%) with Corticobasal Syndrome (CBS).

Of the total sample, 111 (88%) of AD participants, 148 (92%) of CVD participants, 136 (97%) of PD participants, 50 (94%) of FTD participants, and 37 (93%) of ALS participants provided sufficient data for inclusion in this study. Of the 38 excluded participants, 36 were missing NPI-Q scores, and two were missing functional outcomes measures (both basic and instrumental activities of daily living). Of the missing data, three participants (7.8%) had missing data due to verbal refusal to provide data. The remainder of the missing data was for unspecified reasons (i.e., not related to an administrative error, technical challenges, or otherwise). The demographics for included participants are presented in Table 1.

**Table 1.** Baseline Clinical and Demographic Characteristics of Participants in Each Cohort (AD, ALS, FTD, PD, and CVD).

Participant baseline demographics	AD (*n *= 111)	ALS (*n* = 37)	FTD (*n* = 50)	PD (*n* = 136)	CVD (*n* = 148)	*P* value
Sex (%male)	54	62	66	77	68	.002
Age (M ± SD)	71.3 ± 7.9	62.0 ± 8.8	67.7 ± 7.0	67.8 ± 6.3	69.3 ± 7.5	<.001
MoCA	22.7 ± 3.0	25.6 ± 2.9	21.6 ± 3.0	25.9 ± 2.6	25.1 ± 3.0	<.001
ADL % score	98.1 ± 4.7	86.8 ± 14.3	87.9 ± 15.6	96.5 ± 7.4	98.0 ± 5.6	<.001
iADL % score	85.4 ± 17.5	76.9 ± 21.9	60.8 ± 27.5	89.7 ± 14.0	90.6 ± 14.8	<.001
Education(M ± SD)	15.1 ± 3.0	13.8 ± 3.0	13.8 ± 2.6	15.4 ± 2.7	14.6 ± 2.9	<.001
Years in Canada (M ± SD)	63.0 ± 16.5	52.9 ± 13.9	60.6 ± 14.0	58.9 ± 14.6	60.6 ± 15.4	.129
English first language (%)	92	81	90	82	91	.09
Languages spoken (M ± SD)	1.8 ± 0.9	1.7 ± 0.9	1.6 ± 1.0	1.9 ± 1.0	1.8 ± 0.9	.36
Depressive disorder (%)	22.5	18.9	18.0	30.1	22.3	.45
Anxiety disorder (%)	17.1	16.2	10.0	33.8	12.8	.006
Bipolar disorder (%)	0.91	0	0	0	0	
Schizophrenia (%)	0	0	0	0	0	
Stroke (%)	0	0	2.0	1.5	16.9	<.001
LD (%)	2.7	0	4.0	0	0.7	<.001

Abbreviations: LD = Learning disability; MoCA = Montreal Cognitive Assessment; AD = Alzheimer's Disease/Mild Cognitive Impairment; ALS = Amyotrophic lateral sclerosis; FTD = Frontotemporal dementia; PD = Parkinson's disease; CVD = Cerebrovascular disease.

*Note*: ADL score as measured by Physical Self Maintenance Scale (max /24), iADL score as measured by Lawton Instrumental Activities of Daily Living (iADL) scale (max /23). Education (full years of academic coursework, where high school = 12 years, college diploma = 14 years, bachelor's degree = 16 years, master's degree = 18 years, and doctoral degree = 20 years).

Analyses computed using one-way ANOVA or Kruskal–Wallis test as applicable.

### Neuropsychiatric Symptoms

There were significant differences in the NPS frequency among cohorts (χ^2^_(4)_ = 34.4, *p* < .001). Post-hoc, Bonferroni-corrected, pairwise analyses demonstrated that the symptom profile among the FTD cohort differed significantly when compared to AD (χ^2^ = 18.1, *p* < .001), ALS (χ^2^ = 12.7, *p* < .001), CVD (χ^2^ = 17.4, *p* < .001), and PD (χ^2^ = 33.8, *p* < .001) cohorts. Frequencies of individual NPS for each cohort and their comparison across cohorts are shown in Table 2.

**Table 2.** Percentage of Participants in Each Cohort (AD, ALS, FTD, PD, and CVD) Showing Symptoms (Mild, Moderate, or Severe) Evaluated on the NPI-Q.

NPI-Q Symptom	AD (*n* = 126)	ALS (*n* = 40)	FTD (*n* = 53)	PD (*n* = 140)	CVD (*n* = 161)	*P* value*
Delusions	8.7%	2.5%	13.5%	2.9%	7.3%	.06
Hallucinations	4.3%	2.5%	3.8%	9.5%	2.0%	.06
Aggression	28.2%	22.5%	40.4%	17.4%	24.5%	.02
Depression	32.5%	37.5%	37.3%	37.7%	25.8%	.2
Anxiety	25.6%	17.5%	46.2%	21.7%	15.9%	<.001
Euphoria	5.1%	5.0%	15.4%	2.9%	3.3%	.02
Apathy	38.3%	27.5%	56.9%	23.2%	23.2%	<.001
Disinhibition	23.0%	7.5%	43.1%	12.3%	14.6%	<.001
Irritability	37.9%	22.5%	59.6%	27.5%	38.0%	<.001
Motor	12.8%	12.8%	30.8%	5.0%	8.6%	<.001
Night-time behaviours	22.8%	26.3%	53.8%	52.9%	33.6%	<.001
Appetite	28.0%	41.0%	55.8%	27.5%	22.5%	<.001

*P values derived from chi-squared tests.

Abbreviations: NPI-Q = Neuropsychiatric Inventory Questionnaire; AD = Alzheimer's Disease/Mild Cognitive Impairment; ALS = Amyotrophic lateral sclerosis; FTD = Frontotemporal dementia; PD = Parkinson's disease; CVD = Cerebrovascular disease.

Participants with FTD suffered from the greatest frequency of aggression, anxiety, apathy, disinhibition, irritability, euphoria, appetite changes, and motor disturbances. FTD and PD participants showed the greatest frequency of abnormal night-time behaviours. Notably, there were no significant differences among cohorts with respect to the frequency of depression, hallucinations, or delusions. Further details, with included participant counts, are provided in Supplementary Table 1. Participants in the FTD cohort also experienced the greatest overall NPS severity (mean (SD) NPI-Q severity score, AD = 3.7 (4.0), ALS = 3.1 (3.7), FTD = 8.2 (6.2), PD = 3.5 (3.9), CVD = 3.1 (3.9); *H*_(4)_ = 34.5, *p* < .001). Further, partners of participants with FTD reported the greatest degree of overall partner distress (mean (SD) NPI-Q distress score, AD = 4.12 (5.0), ALS = 3.7 (5.3), FTD = 9.1 (9.1), PD = 4.1 (5.7); CVD = 3.5 (5.2), *H*_(4)_ = 21.0, *p* < .001).

### Function

For iADL function, 113 (90%) of AD participants, 40 (100%) of ALS participants, 51 (96%) of FTD participants, 133 (95%) of PD participants, and 149 (93%) of CVD participants were included. There were significant differences among the cohorts on iADL percent scores (mean percent (SD) scores AD = 85.3 (17), ALS = 78.3 (22), FTD = 61.5 (28), PD = 89.7 (14), CVD = 90.8 (14.7); *H*_(4)_ = 69.4, *p* < .001). Post-hoc pairwise comparisons revealed that participants with FTD exhibited greater impairment in iADL scores compared to all cohorts other than ALS (*p* < .001 for all pairwise comparisons).

For ADL function, 115 (91%) of AD participants, 39 (98%) of ALS participants, 52 (98%) of FTD participants, 137 (98%) of PD participants, and 152 (94%) of CVD participants were included. There were significant differences among the cohorts on ADL scores (mean percent (SD) scores, AD = 98.2 (4.6), ALS = 87.5 (14), FTD = 87.8 (16), PD = 96.5(7.3), CVD = 98.1(6), *H*_(4)_ = 94.8, *p* < .001). Post-hoc pairwise comparisons revealed that ALS and FTD cohorts had significantly greater impairment in ADL scores compared to all other cohorts (*p* < .001 for all pairwise comparisons among both groups).

### Relationship between NPS and Function

Across combined participant samples, NPI-Q demonstrated significant negative correlation with MoCA scores (*r_s_* = −0.15, *p* = .001). NPI-Q scores were also negatively correlated with iADL percent scores in AD (*r_s_* = −0.45, *p* < .001), ALS (*r_s_* = −0.52, *p* = .001), FTD (*r_s_* = −0.51, *p* < .001), PD (*r_s_* = −0.38, *p* < .001), and CVD participants (*r_s_* = −0.24, *p* < .01). Further, NPI-Q total score was negatively correlated with ADL percent scores in AD (*r_s_* = −.32, *p *= .001), FTD (*r_s_* = −0.35, *p* = .01), PD (*r_s_* = −0.39, *p* < .001), and CVD (*r_s_* = −0.26, *p* = .002) participants. Among ALS participants, there was no significant correlation between NPI-Q and ADL percent score. Summary correlation data and number of included participants from each cohort are presented in Table 3*.*

**Table 3.** Correlation Matrix Between NPI-Q Total Score with Functional Performance and MoCA Scores Across Cohorts.

		NPI-Q				
		AD	ALS	FTD	PD	CVD
MoCA	Spearman's rho	−0.131	−0.019	−0.189	−0.077	−0.091
	Significance	.169	.911	.188	.370	.279
	N	111	36	50	136	145
iADL percent score	Spearman's rho	−.445	−0.519	−.506	−.376	−.236
	Significance	<.001*	.001*	<.001*	<.001*	.005*
	N	108	37	50	132	143
ADL percent score	Spearman's rho	−.319	−0.094	−.350	−.394	−.260
	Significance	.001*	.587	.013	<.001*	.002*
	N	109	36	50	135	145

*Note: Significant after Bonferonni correctin.

ADL score as measured by Physical Self Maintenance Scale (max /24), iADL score as measured by Lawton Instrumental Activities of Daily Living (iADL) scale (max /23).

Abbreviations: NPI-Q = Neuropsychiatric Inventory Questionnaire; AD = Alzheimer's Disease/Mild Cognitive Impairment; ALS = Amyotrophic lateral sclerosis; FTD = Frontotemporal dementia; PD = Parkinson's disease; CVD = Cerebrovascular disease.

**Figure 1.**
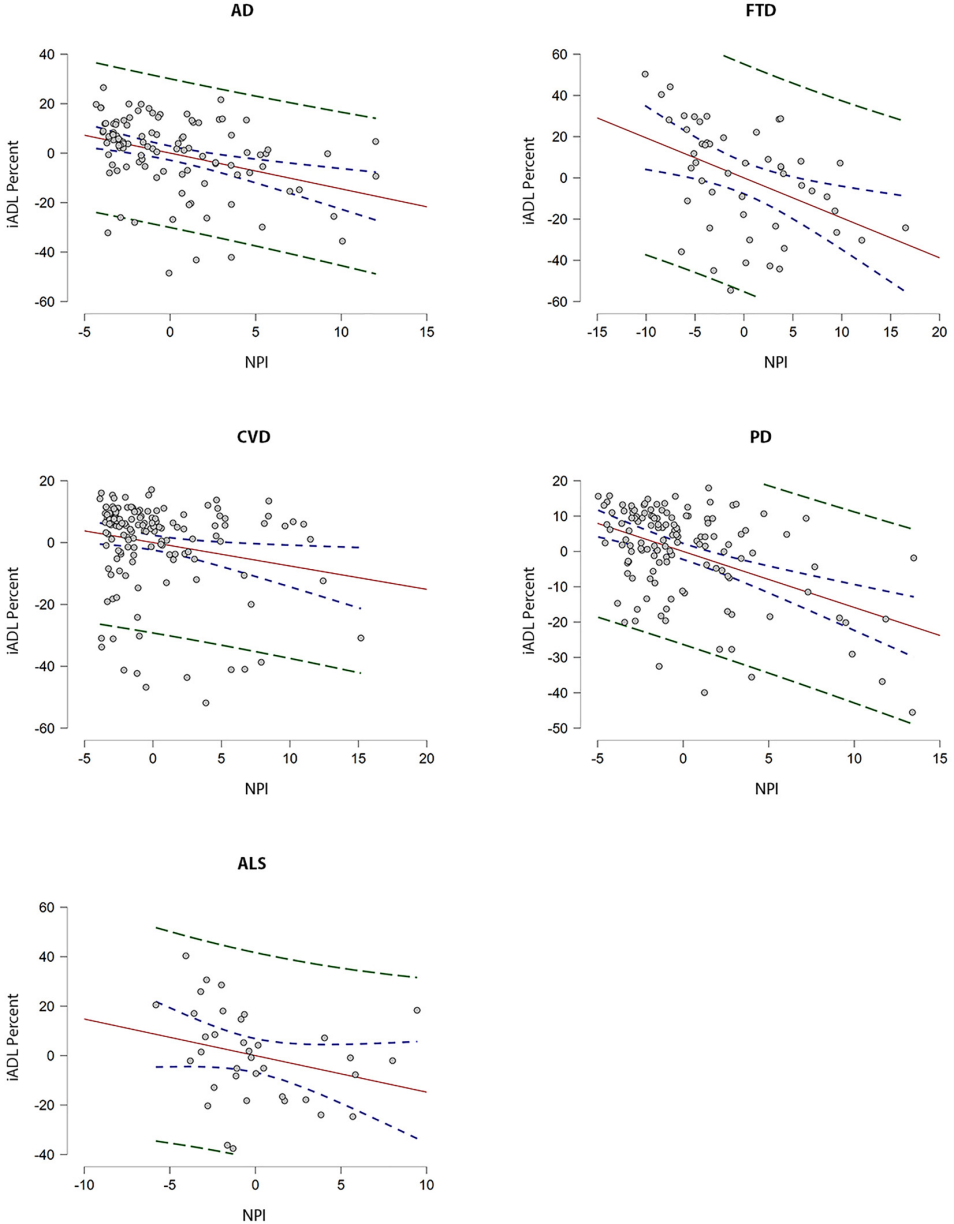
Partial regression plots with *y*-axis representing residuals* from regressing iADL percent score against education, age, and moCA and *x*-axis representing residuals from regressing NPI-Q score against education, age, and MoCA. UPDRS (part 3) residuals are included for the PD cohort. 95% confidence intervals are represented by dotted blue lines and 95% prediction intervals are represented by dotted green lines. The line of best fit shows the strength of the linear relationship between NPI-Q and iADL score among each of the cohorts (AD, FTD, CVD, and PD). *Note. Difference between the observed value of the response variable (iADL score) and the measured value of the response variable predicted from the regression line. iADL score as measured by Lawton instrumental activities of daily living (iADL) scale (max/23). Abbreviations: MoCA = Montreal Cognitive Assessment; NPI-Q = Neuropsychiatric Inventory Questionnaire; FRSR = ALS functional rating scale-revised; UPDRS = Unified Parkinson's Disease Rating Scale; AD = Alzheimer's Disease; ALS = Amyotrophic lateral sclerosis; FTD = Frontotemporal dementia; PD = Parkinson's disease; CVD = Cerebrovascular disease.

With respect to the dependence of iADL on NPI-Q across cohorts, multivariable linear regression did not reveal statistically significant differences (*F*_(4,456)_ = 1.59, *p* = 0.18). Full results of cohort-specific linear regression are presented in Supplementary Tables 2 and 3. Partial plots of NPI-Q residuals against iADL percent scores are shown in Figure 1. In the AD cohort, NPI-Q MoCA, and age contributed to the model (*F*_(4,103)_ = 15.8, *R*^2^ = .36, p < .001). Within the AD cohort, in participants with MCI, MoCA, and age contributed (*F*_(4,64)_ = 6.80, *R*^2^ = .25, *p* < .001), while in participants with dementia, NPI-Q alone contributed to the model (*F*_(4,29)_ = 4.25, *R*^2^ = 0.28, *p* = .008). In the PD cohort, NPI-Q and age contributed to the model (*F*_(5,126)_ = 12.2, *R*^2^ = .30, *p* < .001). In the FTD cohort, NPI-Q alone contributed to the model (*F*_(4,45)_ = 4.3, *R*^2^ = .21, *p* < .01). Similarly, in the CVD cohort, NPI alone contributed to the model (*F*_(4,138)_ = 2.9, *R*^2^ = .05, *p* = .03). In the ALS cohort, FRS-R scores alone contributed to the model (*F*_(5,30)_ = 3.0, *R*^2^ = 0.22, *p* = .03).

**Figure 2.**
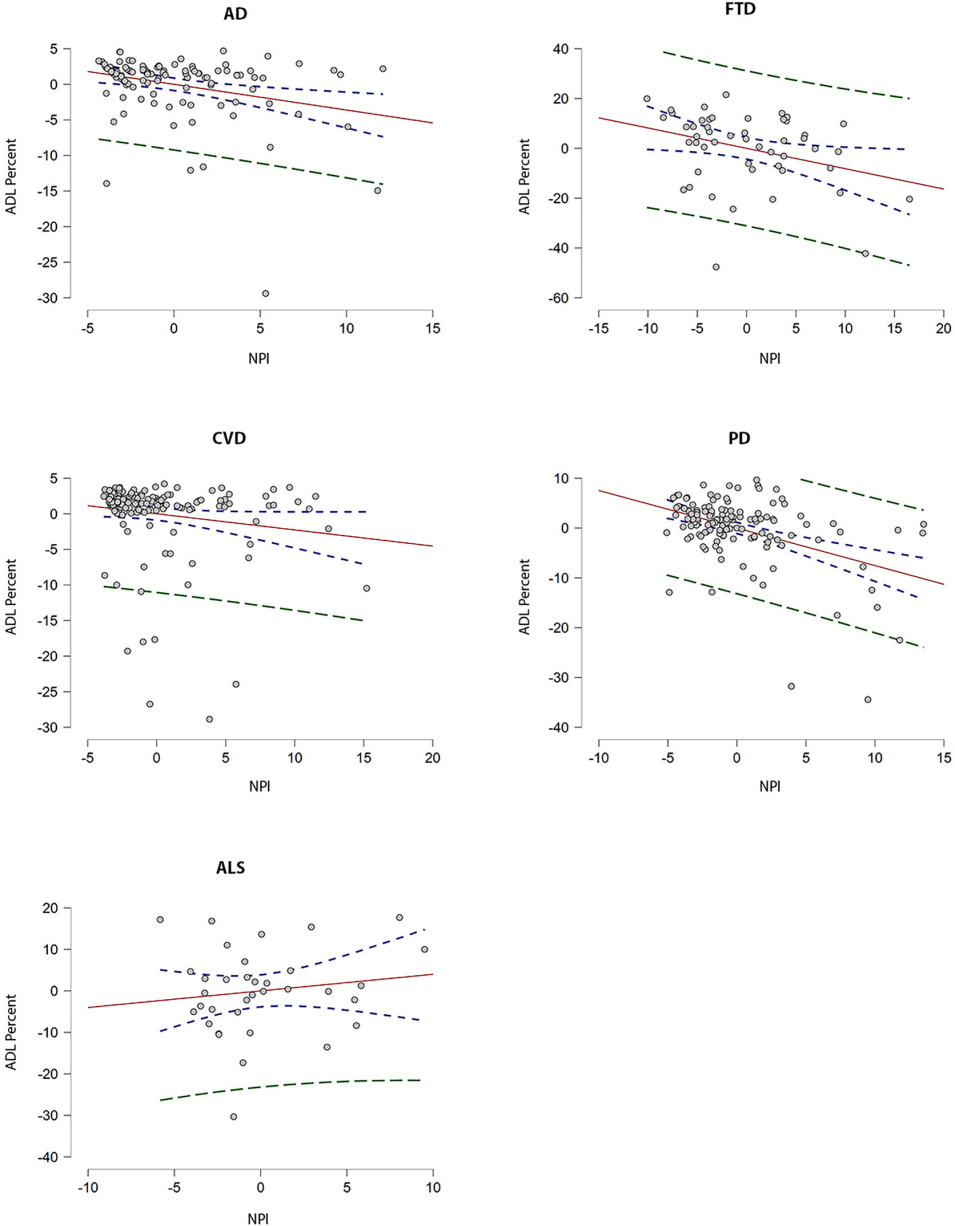
Partial regression plots with *y*-axis representing residuals* from regressing ADL precent score against education, age, and moCA and *x*-axis representing residuals from regressing NPI-Q score against education, age, and MoCA. UPDRS (part 3) residuals are included for the PD cohort. 95% confidence intervals are represented by dotted blue lines and 95% prediction intervals represented by dotted green lines. The line of best fit shows the strength of the linear relationship between NPI-Q and ADL score among each of the cohorts (AD, FTD, CVD, and PD). *Note. Difference between the observed value of the response variable (ADL score) and the measured value of the response variable predicted from the regression line. ADL score as measured by Lawton Instrumental Activities of Daily Living (ADL) scale (max/23). Abbreviations: MoCA = Montreal Cognitive Assessment; NPI-Q = Neuropsychiatric Inventory Questionnaire; FRSR = ALS functional rating scale-revised; UPDRS = Unified Parkinson's Disease Rating Scale; AD = Alzheimer's Disease; ALS = Amyotrophic lateral sclerosis; FTD = Frontotemporal dementia; PD = Parkinson's disease; CVD = Cerebrovascular disease.

With respect to the dependence of ADLs on NPI-Q across cohorts, multivariable linear regression revealed significant differences (*F*_(4,461)_ = 3.1, *p* = .02). Post-hoc pairwise comparisons demonstrated significant differences between the FTD, and ALS (*t* = 2.0, *p* = .03) and CVD (*t* = 2.7, *p* < .01) cohorts. As well, we observed differences between the CVD and PD cohorts (*t* = −2.5, *p* = .01). However, these comparisons did not remain significant following Bonferroni adjustment. Cohort-specific regressions for ADL score revealed significant multivariable models among the AD, FTD, and PD cohorts. Partial plots of NPI-Q residuals against ADL percent score are shown in Figure 2. In the AD cohort, both NPI-Q and age contributed to the model (*F*_(4,104)_ = 4.6, *R*^2^ = .12, *p* = .002). Within the AD cohort, in participants with MCI, NPI-Q alone contributed to the model (*F*_(4,65)_ = 3.4, *R*^2^ = .12, *p* = .02). Similarly, in the FTD cohort, NPI-Q alone contributed to the model (*F*_(4,45)_ = 3.1, *R*^2^ = .14, *p* = .026). In the PD cohort, both NPI-Q and UPDRS contributed to the model (*F*_(5,129)_ = 15.0, *R*^2^ = .34, *p* < .001). In the ALS cohort, FRS-R alone contributed to the model (*F*_(5,29)_ = 5.6, *R*^2^ = 0.40, *p* = .001).

## Discussion

This study conducted a comprehensive evaluation of NPS and their association with function in large, well-defined cohorts of patients across a wide spectrum of neurodegenerative disorders. As hypothesized, we found that the frequency of NPS differed significantly between cohorts, with FTD demonstrating the greatest frequency of NPS. The FTD cohort also had the greatest severity of NPS. Further, there were differences between cohorts in terms of the association between NPS burden and ADLs, and this finding seemed to be largely driven by the FTD cohort. Cohort-stratified analyses revealed a significant association between NPS and activities of living (both basic and instrumental) in AD, FTD, PD, and CVD cohorts. When examined in the presence of covariates (age, education, cognition, and motor function), NPS alone was associated with iADLs in FTD and CVD, whereas NPS, age, and cognition were associated with iADLs in AD. NPS and age were together associated with iADLs in PD. Furthermore, NPS alone were associated with ADLs in FTD and CVD, and together with motor disease burden in PD. Motor disease burden alone was associated with ADLs in ALS. NPS and age were together associated with ADLs in AD.

In line with our work, previous studies have revealed differences in the frequency and severity of NPS among neurodegenerative disease cohorts. In one observational study, participants with FTD had more frequent appetite disturbances, disinhibition, and abnormal motor behaviours as compared to AD and CVD.^26^ This finding is consistent with our data, and in addition, we observed higher frequencies of nearly all other NPS in our FTD cohort as compared to other cohorts. Notably, the previous study had a smaller sample size and higher frequency of overall NPS relative to our sample.^26^ In another large study, FTD was associated with greater overall NPS burden when compared to AD, CVD, and DLB cohorts.^27^ While the authors did not present the frequency of individual NPS within each cohort at baseline,^27^ these findings parallel the results observed in our data. Of note, the behavioural variant subtype was disproportionally represented in our FTD cohort, likely accounting for some of these differences, as previously suggested.^3,27^

### Relationship Between NPS and Function

Interestingly, our data suggested that the dependence of ADL scores on NPS differed significantly between cohorts. While pairwise comparisons did not remain significant after correction, these data support the notion that with greater disease burden, as ADLs become affected, the relationship between NPS and function becomes increasingly clinically relevant. The most notable group differences were observed when comparing FTD cohort to ALS and CVD cohorts.

While cohort-level analyses demonstrated associations between NPS with iADL and ADL across all cohorts, this was not observed in the ALS cohort, possibly due to partial confounding by motor disturbance, consistent with previous data.^28^ Further, our finding in the AD cohort that NPS, cognition, and age together were associated with iADL score, while NPS alone predicted ADL score, suggests that NPS are more closely linked with functional ability at more advanced stages of illness, when basic ADLs become compromised. Additionally, the association of NPS alone with function for both iADL and ADLs in FTD suggests that NPS are the main drivers of functional impairment in FTD throughout the duration of illness. Among PD participants, motor symptom severity demonstrated a significant relationship with ADL scores, but not with iADL scores, suggesting that motor symptoms play a greater role in functional outcomes as disease burden progresses and basic ADLs become compromised. Taken together, these results highlight the need for developing effective clinical interventions for patients experiencing NPS due to neurodegenerative disorders. These should include improved use of existing non-pharmacological and pharmacological approaches, and development of novel therapeutics to improve care for NPS.^1,29,33^

### Limitations

The present study has several limitations. First, this present study was cross-sectional, and we are thus unable to draw conclusions about longitudinal predictions based on our models. Second, the FTD group included participants with bvFTD, PPA, CBS, and PSP, and stratified analyses by these subtypes was not completed due to limited sample sizes. This should be investigated in future studies with larger cohorts. Third, while our diagnostic assessment for patients included standardized criteria and neuroimaging, assessment of biomarkers was not incorporated. Fourth, patients recruited into the study were those with mild-to-moderate illness, potentially reducing the observed range of functional impairment and NPS severity. Therefore, the generalizability of our findings is limited to patients in early or mid-stages of disease. Fifth, unequal cohort sizes included in our data may have resulted in reduced statistical power.

### Conclusion

The present study demonstrates significant differences in the frequency and severity of neuropsychiatric symptoms across neurodegenerative disease cohorts, with the greatest frequency and severity of NPS observed in FTD. Further, NPS burden appears to be associated differently with function across neurodegenerative disorders. These findings elucidate the importance of NPS and their impact on function across the spectrum of neurodegenerative disorders. Considering the irreversibility of cognitive decline, the effective and individualized management of NPS may improve function and other clinical outcomes such as patient and caregiver quality of life.^34^

## Supplemental Material

sj-pdf-1-cpa-10.1177_07067437221147443 - Supplemental material for Neuropsychiatric Symptom Burden across Neurodegenerative Disorders and its Association with Function Click here for additional data file. Supplemental material, sj-pdf-1-cpa-10.1177_07067437221147443 for Neuropsychiatric Symptom Burden across Neurodegenerative Disorders and its Association with Function by Daniel Kapustin, Shadi Zarei, Wei Wang, Malcolm A. Binns, Paula M. McLaughlin, Agessandro Abrahao, Sandra E. Black, Michael Borrie, David Breen, Leanna Casaubon, Dar Dowlatshahi, Elizabeth Finger, Corinne E Fischer, Andrew Frank, Morris Freedman, David Grimes, Ayman Hassan, Mandar Jog, Donna Kwan, Anthony Lang, Brian Levine, Jennifer Mandzia, Connie Marras, Mario Masellis, Joseph B. Orange, Stephen Pasternak, Alicia Peltsch, Bruce G. Pollock, Tarek K. Rajji, Angela Roberts, Demetrios Sahlas, Gustavo Saposnik, Dallas Seitz, Christen Shoesmith, Alisia Southwell, Thomas D.L. Steeves, Kelly Sunderland, Richard H Swartz, Brian Tan, David F. Tang-Wai, Maria Carmela Tartaglia, Angela Troyer, John Turnbull, Lorne Zinman, and Sanjeev Kumar in The Canadian Journal of Psychiatry

sj-docx-2-cpa-10.1177_07067437221147443 - Supplemental material for Neuropsychiatric Symptom Burden across Neurodegenerative Disorders and its Association with Function Click here for additional data file. Supplemental material, sj-docx-2-cpa-10.1177_07067437221147443 for Neuropsychiatric Symptom Burden across Neurodegenerative Disorders and its Association with Function by Daniel Kapustin, Shadi Zarei, Wei Wang, Malcolm A. Binns, Paula M. McLaughlin, Agessandro Abrahao, Sandra E. Black, Michael Borrie, David Breen, Leanna Casaubon, Dar Dowlatshahi, Elizabeth Finger, Corinne E Fischer, Andrew Frank, Morris Freedman, David Grimes, Ayman Hassan, Mandar Jog, Donna Kwan, Anthony Lang, Brian Levine, Jennifer Mandzia, Connie Marras, Mario Masellis, Joseph B. Orange, Stephen Pasternak, Alicia Peltsch, Bruce G. Pollock, Tarek K. Rajji, Angela Roberts, Demetrios Sahlas, Gustavo Saposnik, Dallas Seitz, Christen Shoesmith, Alisia Southwell, Thomas D.L. Steeves, Kelly Sunderland, Richard H Swartz, Brian Tan, David F. Tang-Wai, Maria Carmela Tartaglia, Angela Troyer, John Turnbull, Lorne Zinman, and Sanjeev Kumar in The Canadian Journal of Psychiatry

sj-docx-3-cpa-10.1177_07067437221147443 - Supplemental material for Neuropsychiatric Symptom Burden across Neurodegenerative Disorders and its Association with Function Click here for additional data file. Supplemental material, sj-docx-3-cpa-10.1177_07067437221147443 for Neuropsychiatric Symptom Burden across Neurodegenerative Disorders and its Association with Function by Daniel Kapustin, Shadi Zarei, Wei Wang, Malcolm A. Binns, Paula M. McLaughlin, Agessandro Abrahao, Sandra E. Black, Michael Borrie, David Breen, Leanna Casaubon, Dar Dowlatshahi, Elizabeth Finger, Corinne E Fischer, Andrew Frank, Morris Freedman, David Grimes, Ayman Hassan, Mandar Jog, Donna Kwan, Anthony Lang, Brian Levine, Jennifer Mandzia, Connie Marras, Mario Masellis, Joseph B. Orange, Stephen Pasternak, Alicia Peltsch, Bruce G. Pollock, Tarek K. Rajji, Angela Roberts, Demetrios Sahlas, Gustavo Saposnik, Dallas Seitz, Christen Shoesmith, Alisia Southwell, Thomas D.L. Steeves, Kelly Sunderland, Richard H Swartz, Brian Tan, David F. Tang-Wai, Maria Carmela Tartaglia, Angela Troyer, John Turnbull, Lorne Zinman, and Sanjeev Kumar in The Canadian Journal of Psychiatry
