# Supplementary material for: Neuropsychiatric Symptom Burden across Neurodegenerative Disorders and its Association with Function
Source: Can J Psychiatry. 2023 Jan 13;68(5):347–58. doi: 10.1177/07067437221147443 (PMC10192827; doi:10.1177/07067437221147443)
Supplement: sj-pdf-1-cpa-10.1177_07067437221147443 - Supplemental material for Neuropsychiatric Symptom Burden across Neurodegenerative Disorders and its Association with Function [file sj-pdf-1-cpa-10.1177_07067437221147443.pdf]

NPI Aggression

			No	Yes	Total
Cohort	ADMCI	Count	84	33	117
		Expected Count	88	29	117
	ALS	Count	31	9	40
		Expected Count	30	10	40
	FTD	Count	31	21	52
		Expected Count	39	13	52
	PD	Count	114	24	138
		Expected Count	104	34	138
	CVD	Count	114	37	151
		Expected Count	113	38	151
	Total	Count	374	124	498
		Expected Count	374	124	498

NPI Hallucinations

			No	Yes	Total
Cohort	ADMCI	Count	110	5	115
		Expected Count	109	6	115
	ALS	Count	39	1	40
		Expected Count	38	2	40
	FTD	Count	50	2	52
		Expected Count	49	3	52
	PD	Count	124	13	137
		Expected Count	130	7	137
	CVD	Count	148	3	151
		Expected Count	144	7	151
	Total	Count	471	24	495
		Expected Count	471	24	495

NPI Anxiety

			No	Yes	Total
Cohort	ADMCI	Count	87	30	117
		Expected Count	90	27	117
	ALS	Count	33	7	40
		Expected Count	31	9	40
	FTD	Count	28	24	52
		Expected Count	40	12	52
	PD	Count	108	30	138
		Expected Count	106	32	138
	CVD	Count	127	24	151
		Expected Count	116	35	151
	Total	Count	383	115	498
		Expected Count	383	115	498

NPI Euphoria

			No	Yes	Total
Cohort	ADMCI	Count	111	6	117
		Expected Count	111	6	117
	ALS	Count	38	2	40
		Expected Count	38	2	40
	FTD	Count	44	8	52
		Expected Count	49	3	52
	PD	Count	134	4	138
		Expected Count	131	7	138
	CVD	Count	145	5	150
		Expected Count	142	8	150
	Total	Count	472	25	497
		Expected Count	472	25	497

NPI Apathy

			No	Yes	Total
Cohort	ADMCI	Count	71	44	115
		Expected Count	80	35	115
	ALS	Count	29	11	40
		Expected Count	28	12	40
	FTD	Count	22	29	51
		Expected Count	35	16	51
	PD	Count	106	32	138
		Expected Count	96	42	138
	CVD	Count	116	35	151
		Expected Count	105	46	151
	Total	Count	344	151	495
		Expected Count	344	151	495

NPI disinhibition

			No	Yes	Total
Cohort	ADMCI	Count	90	27	117
		Expected Count	96	21	117
	ALS	Count	37	3	40
		Expected Count	33	7	40
	FTD	Count	29	22	51
		Expected Count	42	9	51
	PD	Count	121	17	138
		Expected Count	113	25	138
	CVD	Count	129	22	151
		Expected Count	123	28	151
	Total	Count	406	91	497
		Expected Count	406	91	497

NPI irritability

			No	Yes	Total
Cohort	ADMCI	Count	72	44	116
		Expected Count	74	42	116
	ALS	Count	31	9	40
		Expected Count	26	14	40
	FTD	Count	21	31	52
		Expected Count	33	19	52
	PD	Count	100	38	138
		Expected Count	88	50	138
	CVD	Count	93	57	150
		Expected Count	96	54	150
	Total	Count	317	179	496
		Expected Count	317	179	496

NPI Motor

			No	Yes	Total
Cohort	ADMCI	Count	102	15	117
		Expected Count	104	13	117
	ALS	Count	34	5	39
		Expected Count	35	4	39
	FTD	Count	36	16	52
		Expected Count	46	6	52
	PD	Count	131	7	138
		Expected Count	122	16	138
	CVD	Count	138	13	151
		Expected Count	134	17	151
	Total	Count	441	56	497
		Expected Count	441	56	497

NPI Nighttime Behaviors

			No	Yes	Total
Cohort	ADMCI	Count	88	26	114
		Expected Count	71	43	114
	ALS	Count	28	10	38
		Expected Count	24	14	38
	FTD	Count	24	28	52
		Expected Count	32	20	52
	PD	Count	65	73	138
		Expected Count	85	53	138
	CVD	Count	99	50	149
		Expected Count	92	57	149
	Total	Count	304	187	491
		Expected Count	304	187	491

NPI Appetite

			No	Yes	Total
Cohort	ADMCI	Count	82	32	114
		Expected Count	80	34	114
	ALS	Count	23	16	39
		Expected Count	27	12	39
	FTD	Count	23	29	52
		Expected Count	36	16	52
	PD	Count	100	38	138
		Expected Count	96	42	138
	CVD	Count	117	34	151
		Expected Count	105	46	151
	Total	Count	345	149	494
		Expected Count	345	149	494

NPI Delusions

			No	Yes	Total
Cohort	ADMCI	Count	105	10	115
		Expected Count	107	8	115
	ALS	Count	39	1	40
		Expected Count	37	3	40
	FTD	Count	45	7	52
		Expected Count	49	3	52
	PD	Count	133	4	137
		Expected Count	128	9	137
	CVD	Count	140	11	151
		Expected Count	141	10	151
	Total	Count	462	33	495
		Expected Count	462	33	495

NPI Depression

			No	Yes	Total
Cohort	ADMCI	Count	79	38	117
		Expected Count	79	38	117
	ALS	Count	25	15	40
		Expected Count	27	13	40
	FTD	Count	32	19	51
		Expected Count	34	17	51
	PD	Count	86	52	138
		Expected Count	93	45	138
	CVD	Count	112	39	151
		Expected Count	101	50	151
	Total	Count	334	163	497
		Expected Count	334	163	497

Abbreviations:

NPI: Neuropsychiatric Inventory Questionnaire
AD/MCI: Alzheimer's Disease / Mild Cognitive Impairment
ALS: Amyotrophic lateral sclerosis
FTD: Frontotemporal dementia
PD: Parkinson's disease
CVD: Cerebrovascular disease

Supplementary Material 1. Chi-square or Fisher's exact test (expected cell count less than five) were used to compare the frequency of individual NPS across each cohort. The data displayed demonstrates the observed and predicted cell counts for the presence ("Yes") or absence ("No") of individual symptoms for participants in each group.
