# Supplementary material for: Neuropsychiatric Symptom Burden across Neurodegenerative Disorders and its Association with Function
Source: Can J Psychiatry. 2023 Jan 13;68(5):347–58. doi: 10.1177/07067437221147443 (PMC10192827; doi:10.1177/07067437221147443)
Supplement: sj-docx-2-cpa-10.1177_07067437221147443 - Supplemental material for Neuropsychiatric Symptom Burden across Neurodegenerative Disorders and its Association with Function [file sj-docx-2-cpa-10.1177_07067437221147443.docx]

|  | | | | | | |
| --- | --- | --- | --- | --- | --- | --- |
| **ADMCI** | |  | | **Standardized Beta** | **t** | **p** |
|  |  | Education Level |  | 0.124 | 1.57 | 0.12 |
|  |  | Age |  | -0.174 | -2.23 | 0.03 |
|  |  | MoCA |  | 0.409 | 5.14 | < .001 |
|  |  | NPI total |  | -0.316 | -4.03 | < .001 |
| **ALS** | |  | |  |  |  |
|  |  | Education Level |  | 0.138 | 0.88 | 0.38 |
|  |  | Age |  | 0.04 | 0.25 | 0.81 |
|  |  | MoCA |  | 0.197 | 1.21 | 0.24 |
|  |  | NPI total |  | -0.249 | -1.62 | 0.12 |
|  |  | ALS FRSR Total |  | 0.43 | 2.71 | 0.01 |
| **FTD** | |  | |  |  |  |
|  |  | Education Level |  | 0.031 | 0.24 | 0.81 |
|  |  | Age |  | -0.115 | -0.89 | 0.38 |
|  |  | MoCA |  | 0.196 | 1.49 | 0.14 |
|  |  | NPI total |  | -0.437 | -3.28 | 0.002 |
| **PD** | |  | |  |  |  |
|  |  | Education Level |  | -0.011 | -0.15 | 0.88 |
|  |  | Age |  | -0.286 | -3.62 | < .001 |
|  |  | MoCA |  | 0.125 | 1.59 | 0.11 |
|  |  | NPI total |  | -0.432 | -5.79 | < .001 |
|  |  | UPDRS Part 3 total |  | -0.07 | -0.91 | 0.37 |
| **CVD** | |  | |  |  |  |
|  |  | Education Level |  | -0.007 | -0.09 | 0.93 |
|  |  | Age |  | -0.065 | -0.76 | 0.45 |
|  |  | MoCA |  | 0.122 | 1.42 | 0.16 |
|  |  | NPI total |  | -0.224 | -2.71 | 0.01 |
| **AD** | |  | |  |  |  |
|  |  | Education Level |  | 0.133 | 0.87 | 0.39 |
|  |  | Age |  | -0.304 | -2.05 | 0.05 |
|  |  | MoCA |  | 0.236 | 1.55 | 0.13 |
|  |  | NPI total |  | -0.433 | -2.93 | 0.01 |

Note. These regressions included 107 (85%) AD participants, 35 (88%) ALS participants, 50 (94%) FTD participants, 132 (94%) PD participants, and 143 (89%) CVD participants. ADL score as measured by Physical Self Maintenance Scale (max /24), iADL score as measured by Lawton Instrumental Activities of Daily Living (iADL) scale (max /23). Education (full years of academic coursework, where high school = 12 years, college diploma = 14 years, bachelor’s degree = 16 years, master’s degree = 18 years, and doctoral degree = 20 years).

Abbreviations:

MoCA: Montreal Cognitive Assessment

NPI total: Neuropsychiatric Inventory Questionnaire (total score)

FRSR: ALS functional rating scale-revised

UPDRS: Unified Parkinson's Disease Rating Scale

AD/MCI: Alzheimer’s Disease / Mild Cognitive Impairment

ALS: Amyotrophic lateral sclerosis

FTD: Frontotemporal dementia

PD: Parkinson’s disease

CVD: Cerebrovascular disease

**Supplementary Table 2.** *Multiple linear regression models evaluating the relationship between NPI total score, Age, Education, and MoCA (independent variables) and iADL percent score (dependent variable) across participant cohorts (AD/MCI, ALS, FTD, PD, and CVD).*
