# Supplementary material for: Neuropsychiatric Symptom Burden across Neurodegenerative Disorders and its Association with Function
Source: Can J Psychiatry. 2023 Jan 13;68(5):347–58. doi: 10.1177/07067437221147443 (PMC10192827; doi:10.1177/07067437221147443)
Supplement: sj-docx-3-cpa-10.1177_07067437221147443 - Supplemental material for Neuropsychiatric Symptom Burden across Neurodegenerative Disorders and its Association with Function [file sj-docx-3-cpa-10.1177_07067437221147443.docx]

|  | | | | | | | |
| --- | --- | --- | --- | --- | --- | --- | --- |
|  | | | | | | | |
| **ADMCI** | |  | | **Standardized Beta** | | **t** | **p** |
|  |  | Education Level |  | 0.081 | | 0.88 | 0.38 |
|  |  | Age |  | -0.188 | | -2.05 | 0.04 |
|  |  | MoCA |  | 0.017 | | 0.19 | 0.85 |
|  |  | NPI total |  | -0.306 | | -3.35 | 0.001 |
| **ALS** | |  | |  |  |  |  |
|  |  | Education Level |  | 0.23 | | 1.66 | 0.11 |
|  |  | Age |  | 0.136 | | 0.95 | 0.35 |
|  |  | MoCA |  | 0.08 | | 0.55 | 0.59 |
|  |  | NPI total |  | 0.104 | | 0.76 | 0.45 |
|  |  | ALS FRSR Total |  | 0.71 | | 5.08 | < .001 |
| **FTD** | |  | |  |  |  |  |
|  |  | Education Level |  | 0.116 | | 0.87 | 0.39 |
|  |  | Age |  | -0.153 | | -1.13 | 0.27 |
|  |  | MoCA |  | 0.192 | | 1.40 | 0.17 |
|  |  | NPI total |  | -0.324 | | -2.33 | 0.02 |
| **PD** | |  | |  |  |  |  |
|  |  | Education Level |  | -0.054 | | -0.76 | 0.45 |
|  |  | Age |  | -0.135 | | -1.80 | 0.07 |
|  |  | MoCA |  | 0.06 | | 0.80 | 0.43 |
|  |  | NPI total |  | -0.399 | | -5.58 | < .001 |
|  |  | UPDRS Part 3 total |  | -0.349 | | -4.76 | < .001 |
| **CVD** | |  | |  |  |  |  |
|  |  | Education Level |  | 0.119 | | 1.44 | 0.15 |
|  |  | Age |  | -0.146 | | -1.70 | 0.09 |
|  |  | MoCA |  | -0.05 | | -0.58 | 0.57 |
|  |  | NPI total |  | -0.162 | | -1.96 | 0.05 |
| **AD** | |  | |  |  |  |  |
|  |  | Education Level |  | 0.076 | | 0.45 | 0.66 |
|  |  | Age |  | -0.371 | | -2.22 | 0.03 |
|  |  | MoCA |  | -0.017 | | -0.10 | 0.92 |
|  |  | NPI total |  | -0.233 | | -1.40 | 0.17 |

Note. These regressions included 107 (85%) AD participants, 35 (88%) ALS participants, 50 (94%) FTD participants, 132 (94%) PD participants, and 143 (89%) CVD participants. ADL score as measured by Physical Self Maintenance Scale (max /24), iADL score as measured by Lawton Instrumental Activities of Daily Living (iADL) scale (max /23). Education (full years of academic coursework, where high school = 12 years, college diploma = 14 years, bachelor’s degree = 16 years, master’s degree = 18 years, and doctoral degree = 20 years)

Abbreviations:

MoCA: Montreal Cognitive Assessment

NPI total: Neuropsychiatric Inventory Questionnaire (total score)

FRSR: ALS functional rating scale-revised

UPDRS: Unified Parkinson's Disease Rating Scale

AD/MCI: Alzheimer’s Disease / Mild Cognitive Impairment

ALS: Amyotrophic lateral sclerosis

FTD: Frontotemporal dementia

PD: Parkinson’s disease

CVD: Cerebrovascular disease

**Supplementary Table 3.** *Multiple linear regression models evaluating the relationship between NPI total score, Age, Education, MoCA, ALS FRS-R, and UPDRS part 3 (independent variables) and ADL percent score (dependent variable) across participant cohorts (AD/MCI, ALS, FTD, PD, and CVD).*
